# A novel evaluation tool for combat trauma simulation: Delphi expert panel consensus report

**DOI:** 10.1186/s13037-026-00493-z

**Published:** 2026-06-26

**Authors:** Samuel Weber, Henrik Teuber, Basil Andreas Hatz, Michael Kuny, Bastian Grande, Michaela Kolbe, Hans-Christoph Pape, Sascha Jan Baettig

**Affiliations:** 1 Faculty of Medicine, University of Zürich, Zurich, Switzerland; 2 Department of Traumatology, University Hospital Zurich, Zurich, Switzerland; 3 Swiss Armed Forces Medical Service, Bern, Switzerland; 4 Department of Orthopaedics and Traumatology, University Hospital Basel, Basel, Switzerland; 5 Institute of Anaesthesiology and Perioperative Medicine, University Hospital Zurich, Zurich, Switzerland; 6 Simulation Centre, University Hospital Zurich, Zurich, Switzerland

**Keywords:** Combat injuries, Simulation-based education, Trauma management, Performance assessment tool, Civil-military collaboration

## Abstract

**Background:**

Modern armed conflicts reveal increasingly complex injury patterns that challenge both civilian and military healthcare systems. In Switzerland, exposure to such injuries is limited. For a planned multicenter, high-fidelity, in-situ simulation-based study assessing trauma care performance across both military and civilian prehospital and clinical teams, a standardized and validated evaluation tool is required. The current study was designed to develop a consensus-based performance assessment tool/framework for evaluating the management of complex combat trauma, integrating civilian and military standards of care.

**Methods:**

A structured consensus process was conducted, combining an initial nominal group technique with a multi-round Delphi process. Fourteen experts from military medicine, emergency medicine, anesthesiology, and trauma surgery participated. Assessment items were iteratively refined until predefined consensus criteria were achieved.

**Results:**

Consensus was reached after five Delphi rounds. The evaluation tool includes 35 items covering key domains such as prioritization of interventions, procedural quality, adherence to trauma principles, and time to critical actions. Initial differences between civilian and military perspectives—particularly regarding cervical spine immobilization, neurological assessment, coagulation management, and fluid resuscitation—were identified and resolved through the consensus process.

**Conclusion:**

The results of this Delphi-based evaluation framework highlight both areas of consensus and ongoing controversy in modern combat trauma care. Differences between civilian and military perspectives are largely driven by divergent injury patterns, resources, and operational constraints. By explicitly incorporating these differences into a standardized evaluation tool, this study provides a robust framework for comparing performance across different levels of trauma care.

**Supplementary Information:**

The online version contains supplementary material available at https://doi.org/10.1186/s13037-026-00493-z.

## Introduction

Modern armed conflicts have highlighted the evolving nature of battlefield injuries and the increasing complexity of casualties presenting to healthcare providers [1, 2]. Explosive mechanisms, now responsible for the majority of combat injuries, result in complex, multifaceted trauma patterns ranging from primary blast injuries to quaternary effects such as thermal burns and crush injuries [1, 3]. Further compounding these challenges, limited evacuation—particularly by air—leads to prolonged prehospital care lasting hours or days, requiring military and prehospital teams to deliver more advanced and sustained care beyond their traditional scope [2, 4, 5]. In addition, differences in terminology, abbreviations, training paradigms, and clinical algorithms between military and civilian systems may further hinder communication and coordination across levels of care [6–8]. 

While the Swiss healthcare system is highly advanced, both civilian and military teams have limited practical experience managing such combat-related injury patterns under realistic operational conditions [9]. For example in European Level 1 trauma centers, the vast majority of injuries result from isolated blunt mechanisms, such as falls or motor vehicle accidents [10]. Consequently, exposure to complex penetrating and blast-related trauma remains rare, potentially leading to gaps in preparedness that may adversely affect both patient outcomes and provider safety [11]. Simulation-based training has emerged as a valuable approach to address this gap in exposure, particularly for rare but high-acuity scenarios [8, 12]. Moreover, in modern conflicts, the boundaries between military and civilian systems, as well as prehospital and in-hospital care, are increasingly blurred, requiring that initial stabilization and life-saving interventions be delivered effectively across all levels of care [13]. 

We therefore developed a multicenter, high-fidelity, in-situ simulation study to evaluate the management of a severely injured combat casualty across different levels of both civilian and military trauma care. Meaningful comparison of team performance requires a robust, structured, and context-specific assessment tool that reflects both civilian and military standards of care. Existing instruments were considered insufficient to capture the key principles of early management in penetrating multisystem trauma, particularly regarding clinical depth and contextual specificity.

Beyond enabling comparison, such a tool is essential to objectively assess performance and learning outcomes in simulation-based combat trauma training. While the long-term goal is inter-team comparison, the immediate need is a reliable framework to measure performance in complex simulation environments. Although developed for a specific scenario, the tool can be adapted with minimal modifications to similar trauma cases, supporting broader applicability.

Such comparison, particularly across different levels of care and professional backgrounds, requires a high degree of standardization. This applies not only to the simulation design and delivery, but also to the framework used to assess medical performance. However, military, prehospital, and in-hospital treatment paradigms differ substantially in terms of prioritization, clinical decision-making, and therapeutic strategies [6, 14]. As a result, experts from these domains often emphasize different aspects of care, which complicates objective and reproducible performance assessment.

To address these challenges, it is essential to first identify areas of divergence and subsequently establish a shared framework that defines common standards across key domains, including prioritization of interventions, quality and type of care, time to completion of critical actions, and team interaction. The aim of this study was to develop a consensus-based, standardized performance evaluation tool using a structured Delphi process, designed to enable objective assessment and comparison of performance across military and civilian settings in a complex combat trauma simulation.

## Methods

### Study design

To inform future research on combat trauma management and enable standardized evaluation of treatment and team performance in high-risk scenarios, we applied a structured, stepwise approach. First, a representative combat-related polytrauma pattern was identified based on contemporary injury epidemiology, predominantly driven by high-energy blast mechanisms.

Second, a high-fidelity simulation scenario was developed based on this injury pattern, incorporating key features such as multiple hemorrhages, airway compromise, thoracic injury and burns, while ensuring realism and reproducibility across different care settings. Although the tactical environment is adapted to each participating team’s operational context, the underlying medical scenario remains strictly standardized across all sites. Each simulation is conducted by a minimum of three healthcare professionals and has a fixed duration of 25 min (see Appendix 1 for details).

Third, a structured consensus process was performed to define performance metrics and establish target time frames for critical interventions. The development of our case-specific evaluation framework followed a structured consensus methodology combining the Nominal Group Technique and a multi-round Delphi process. The Delphi process was conducted in accordance with the DELPHISTAR recommendations and is reported accordingly [15]. 

### Nominal group technique

The Nominal Group Technique (NGT) is a structured, face-to-face consensus method designed to facilitate idea generation and prioritization through equal participation and moderated discussion [16]. The classical NGT comprises four sequential stages: Silent idea generation, round-robin sharing of ideas without discussion, clarification and structured group discussion, and voting and ranking to achieve initial consensus [17]. 

In this study, the full NGT process was systematically applied as the initial step in developing the assessment framework. A dedicated session was conducted with 5 of the 14 experts, all of whom were board-certified physicians in anesthesiology or trauma surgery and/or active-duty military physicians within the Swiss Armed Forces or with equivalent experience.

During the silent generation phase, participants independently generated potential assessment items based on the predefined simulation scenario. This was followed by a structured round-robin phase, in which each participant presented their items sequentially without interruption. All proposed items were then subjected to clarification and moderated discussion, allowing refinement, consolidation, and removal of redundancies. Finally, items were prioritized through a voting process, resulting in an initial, consensus-based draft of the evaluation framework. The resulting preliminary framework comprised the evaluation of all scenario-specific items addressing critical interventions and procedural quality and was aligned with established guidelines, including Advanced Trauma Life Support (ATLS), Prehospital Trauma Life Support (PHTLS), and Tactical Combat Casualty Care (TCCC). No time-based performance targets were defined at this stage.

### Delphi consensus process

The initial expert panel was expanded to 14 participants. The panel was deliberately chosen to ensure balanced representation of both civilian and military medical institutions across Switzerland, with seven experts from each domain.

Eligibility criteria for civilian experts included substantial clinical experience as physicians in emergency medicine, anesthesiology, or trauma surgery, or equivalent expertise in prehospital care as paramedics. Military experts comprised recruited from active-duty military physicians and master medics within the professional armed forces, including personnel with advanced training such as Special Forces Medical Sergeants. Following panel selection, participants were introduced to the study objectives and the standardized simulation scenario.

The Delphi method, originally developed by the RAND Corporation, is a systematic approach for achieving consensus on complex problems, particularly in situations with limited empirical evidence [18]. Its core principles include participant anonymity, iterative rating rounds with controlled feedback, and statistical aggregation of group responses.

The preliminary assessment tool developed via NGT was subsequently subjected to a multi-round Delphi process. In the first round, experts were presented with the simulation scenario and the initial draft of the evaluation form. They were asked to assign relative importance to each item using point values from 1 to 30 and to define acceptable and realistic time frames for completion of each critical medical intervention. Experts were also invited to propose modifications to existing items, suggest deletions, or recommend additions.

In subsequent rounds, experts received anonymized aggregated results from the previous round and were given the opportunity to reconsider and adjust their ratings considering clinical guidelines and best practices. Comparative pairwise ranking of individual items was deliberately avoided, as such comparisons do not accurately reflect real-world clinical practice. The goal was to define the optimal achievable performance of a treating medical team.

The Delphi process was conducted electronically in written form, with clearly defined deadlines for completion of each round. Consensus was predefined as the point at which no further amendments, additions, or deletions to the evaluation form were proposed by the expert panel.

### Ethics committee approval

This study does not involve research on human patients, identifiable human data, or human tissue. According to Swiss national regulations and the confirmation of the Cantonal Ethics Committee Zurich, the project does not fall within the scope of the Swiss Human Research Act. The Ethics Committee has therefore granted a waiver for this study (Req-2025-01335).

### Statistical analysis

Descriptive analyses were performed to explore potential differences in expert opinions between subgroups (military vs. civilian) and across items or thematic domains. Data analysis was conducted using IBM SPSS Statistics, Version 29 (IBM Corporation, Armonk, NY, USA).

## Results

The expert panel comprising a total of fourteen members: seven military experts from the Swiss Armed Forces and seven civilian trauma-specialized experts. Eight panelists were physicians with specializations in anesthesiology, trauma surgery, intensive care, or orthopedics, while the remaining six were non-physician experts, including paramedics and Special Forces Medical Sergeants (18D). All panelists participated in all five Delphi rounds, with the entire process spanning 120 days. Consensus was reached after the fifth round, and the evaluation form was formally approved by all members. A detailed summary of all Delphi rounds is provided in Appendix 2.

The assessment tool consists of eight sections with a total of 35 items (Table 1). Each item is assigned a relative weight between 1 and 30 points for intervention and quality, along with a predefined list of point deductions for errors and, where applicable, a target time. Successfully completing an intervention within the set time goal awards 10 points per item. If the time goal is exceeded, 1 point is deducted per minute of delay, or 0 points if the intervention is not performed or occurs more than 10 min after the target time. In total, the tool allows for 1,061 points, of which 300 points are allocated for timing and 761 points for intervention quality.

**Table 1:** Final assessment tool

Therapeutic Measure (Yes/No, if applicable: which measure and any problems)		Time Requirement (0–10 Points) [min]	Quality – Therapeutic Measure [1–30 Points]
**1 General / Examination**		**Max. Points: 60**	**Max. Points: 154**
1.1	**Finding / Removal of dangerous objects** (2x hand grenades, 1x pistol, 2x magazine); 5 pts per removed item	2	25
1.2	**Physical examination / Undressing** Groin & axilla / extremities / head / buttocks / back, hands & feet & torso; – 5 pts per clothing item not removed or region not examined	7	25
1.3	**Primary Survey** Based on clearly identifiable treatment priority (XABCD, MARCH); –5 pts per missing organ system, − 3 pts per inappropriate prioritization	6	27
1.4	**Recording of relevant vital parameters** (HR, SpO₂, BP, Pulse, Capillary Refill, Temperature, RR); –5 pts for each vital parameter not recorded/examined	8	26
1.5	**Heat preservation** Maximal points: Active warming (fluid warmer, active heating blankets / heaters), passive measures (warming blankets / emergency rescue foil) max. 10 pts; –2 pts per 15 s of unnecessary uncovering	8	21
1.6	**Log Roll** Maximal points: Visual and tactile injury search (entry or exit wounds, spinal fractures). −5 points if visual only.	9	18
1.7	**Aseptic technique** –2 pts per clear sterility error (surgical measures, but also dressings / wound packing, IV line)		12
**2 Exsanguinating Bleeding**		**Max. Points: 30**	**Max. Points: 83**
2.1	**Treatment of 1st massive hemorrhage – Right thigh** Tourniquet application: − 5 pts each for misplacement, insufficient pre-tightening/tightening, not applied on correct spot (skin, 5–8 cm proximal of the wound or on a joint) Wound packing / pressure dressing: − 5 pts for insufficient pressure, wrong material, not on skin, inadequate fixation	2	28
2.2	**Treatment of 2nd massive hemorrhage – Right buttock** Tourniquet application: − 5 pts each for misplacement, insufficient pre-tightening/tightening, not applied on correct spot (skin, 5–8 cm proximal of the wound or on a joint) Wound packing / pressure dressing: − 5 pts for insufficient pressure, wrong material, not on skin, inadequate fixation	3	28
2.3	**Treatment of 3rd massive hemorrhage – Left upper arm** Tourniquet application: − 5 pts each for misplacement, insufficient pre-tightening/tightening, not applied on correct spot (skin, 5–8 cm proximal of the wound or on a joint) Wound packing / pressure dressing: − 5 pts for insufficient pressure, wrong material, not on skin, inadequate fixation	3	27
**3 Airway**		**Max. Points: 30**	**Max. Points: 90**
3.1	**Patient positioning** Maximal points: Leaning forward or lateral position; 10 pts for 30° elevated, sitting or lateral position/recovery position	4	21
3.2	**Surgical cricothyrotomy** Progressing airway obstruction after 10 min in supine position or after 20 min in lateral / seated position; –5 pts per clear error: disinfection, local anaesthesia/anaesthesia, incision, open surgical technique/bougie-aided technique, suction through the cannula, cuff with correct pressure		28
3.3	**Position check of surgical airway** Maximal points: Continuous etCO₂, clinical check (auscultation / sonography / tactile); etCO₂ only: 10 pts, clinical check only: 0 pts	4	26
3.4	**Cervical spine immobilization** Maximal points: Continuous in line cervical spine stabilization. −5 pts for any patient movement or manipulation without stabilization.	6	15
**4 Breathing**		**Max. Points: 50**	**Max. Points: 107**
4.1	**Clinical thorax examination** –3 pts per missing item (expose chest, visual injury assessment, tactile injury assessment, auscultation, assessment of tracheal asymmetry, assessment of chest rise asymmetry, assessment for rib fractures)	7	22
4.2	**Oxygen therapy** Maximal points: 12 L via reservoir mask or similar measures	5	25
4.3	**Instrumental thorax examination** Ultrasound / chest X-ray: maximum points for correctly identifying a haemato-pneumothorax and, combined with clinical findings, deriving the correct therapy	14	15
4.4	**Basic therapy** (Occlusive dressing, chest seal, needle decompression) Max points: Correct application of a chest seal or similar device and, if indicated, needle decompression; −5 pts for clear errors (e.g., improper chest seal fixation, incorrect decompression site, or improper needle use).	7	23
4.5	**Advanced surgical therapy** Chest drain, finger thoracostomy, thoracotomy; –5 pts per error: disinfection, local anaesthesia/anaesthesia, correct localization, correct surgical technique, correct fixation, position check	14	22
**5 Circulation**		**Max. Points: 80**	**Max. Points: 180**
5.1	**Re-evaluation of haemorrhage control measures** Bleeding control, if applicable: tourniquet approximation / repositioning / conversion Conversion of a tourniquet in the presence of haemorrhagic shock is a clear error and scored 0 points. -5 pts if tourniquets are not approximated and applied at the correct location and skin level.	9	24
5.2	**Recognition of haemorrhagic shock** Maximal points: Diagnosis must be verbalized or clear therapeutic measures are initiated	6	27
5.3	**Pelvic binder** Indication: pain, crepitation, haemorrhagic shock; -13 pts if pelvic belt applied over uniform, -10 pts for incorrect position, especially too high.	7	23
5.4	**Venous access** –5 pts per error: disinfection, minimum 2 × 18G access, sterile connection, fixation, function check	7	24
5.5	**Adequate fluid therapy** Includes fluid administration per institutional standards, including blood products and volume therapy with NaCl or balanced electrolyte solutions.	10	23
5.6	**Adequate blood pressure management** Permissive hypotension with target BP 80–90 mmHg systolic or 50–60 mmHg MAP; − 3 pts for each minute beyond the time requirement during which target BP is not achieved	13	22
5.7	**Correct handling of packed red blood cells (pRBC)** –3 pts per error: ABO check on pRBC, visual check (clots), expiry date check, use of transfusion set, depending on local protocol: crossmatch, re-assessment, administration of 2.25 mmol calcium gluconate	14	17
5.8	**Extended assessment of bleeding sources** Maximal points: eFAST + clinical examination (abdomen, femur, pelvis, head); max. 10 pts if only clinical examination is performed	15	18
**6 Disability**		**Max. Points: 30**	**Max. Points: 81**
6.1	**Assessment of central neurological status** Correct GCS = 20 pts, assessment using AVPU only: 10 pts	7	20
6.2	**Examination for peripheral neurological deficits** e.g. paraplegia	12	17
6.3	**Adequate Analgesia** Dosages: Fentanyl 100–150 µg iv. / 400–800 µg po., Ketamine 25–50 milligram iv. -5 pts for underdosing, -10 pts or more for overdosing	13	22
6.4	**Adequate Anesthesia** Adequate induction and maintenance. Dosages: 5–15 milligram Midazolam iv, Propofol 150–300 milligram/hour iv., Sevoflurane < MAC 0.7 -5 pts for underdosing, -10 pts or more for overdosing		22
**7 Coagulation Management**		**Max. Points: 10**	**Max. Points: 35**
7.1	**TXA (Tranexamic Acid)** Dosages per applicable guidelines: Civilian 1 gram, Military 2 g -5 pts if patient is awake and no antiemetic given	9	20
7.3	**Fibrinogen** Bonus points! Empirically 2–6 g iv. or based on point-of-care coagulation analysis (ROTEM/TEG)		15
**8 Other**		**Max. Points: 10**	**Max. Points: 31**
8.1	**Treatment / Wound care for burns**	16	16
8.2	**Antibiotic therapy**	17	15

Abbreviations: Pts: Points; HR: Heart Rate; BP: Blood Pressure; RR: Respiratory Rate; etCO₂: End Tidal Carbon Dioxide; 18G: 18 Gauge; pRBC: Packed Red Blood Cells; GCS: Glasgow Coma Scale; IV: Intravenous; MAC: Minimal Alveolar Concentration; ROTEM: Rotational Thromboelastometry, TEG: Thromboelastography

Assessment of behavioral and human factors was not part of the Delphi process, as these will be evaluated separately during the simulation study using the T-NOTECHS score [19] and an adapted 1–5 Likert scale based on the CRM criteria by Lei and Palm [20]. 

During the Delphi process, several content adaptations were made to the assessment framework. A summary of all modifications and discussions is provided in (Appendix 3). The most debated section concerned initial fluid resuscitation, including choice of fluids, timing, prioritization relative to hemorrhage control, and technical aspects of transfusion. Other key areas of discussion included early coagulation monitoring and therapy, cervical spine immobilization, neurological examination, and removal of weapons.

**Fig. 1:**
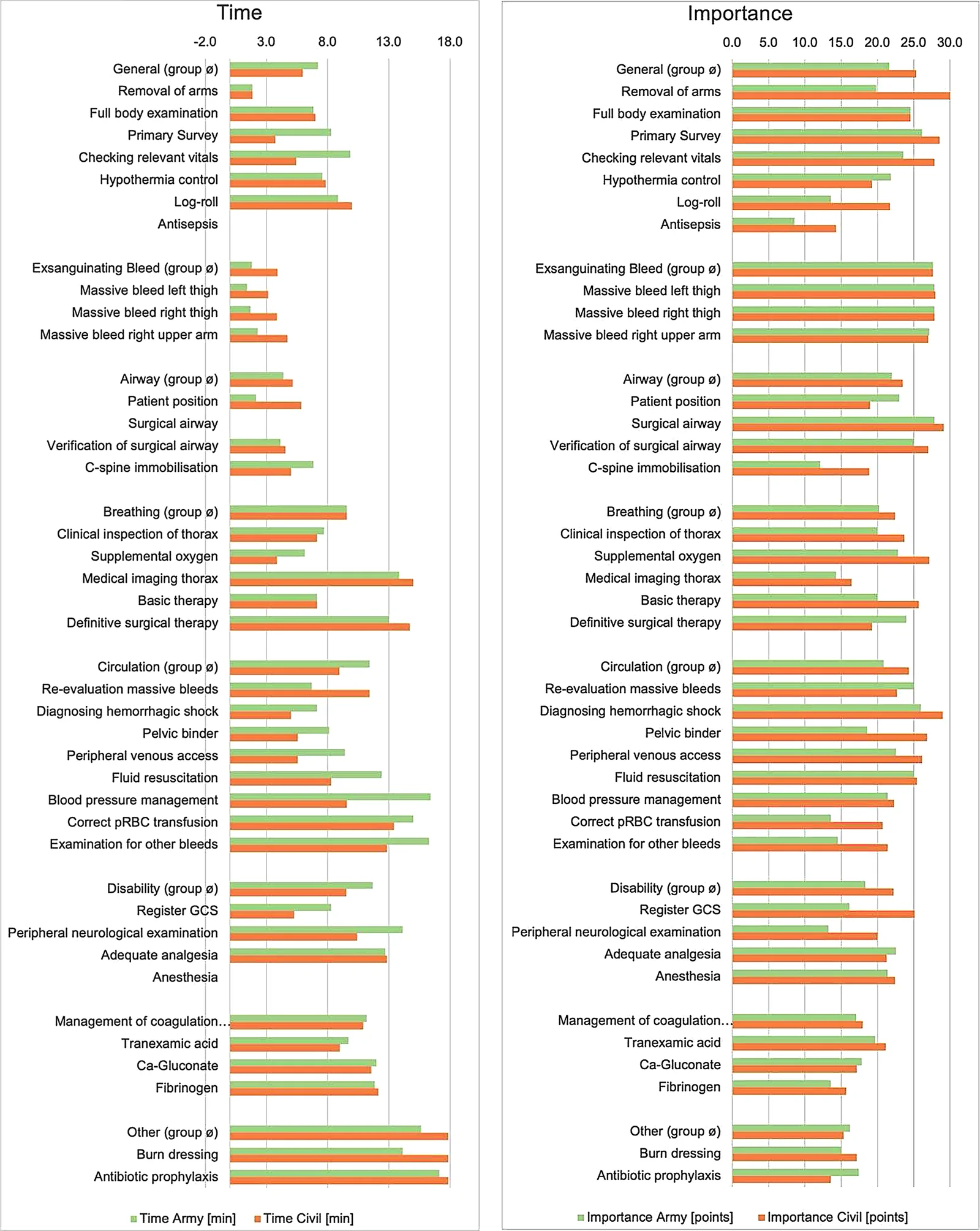
Comparison of summarized ratings between military (green) and civilian experts (orange), showing both intervention quality and timing performance

Overall, experts gave comparable scoring recommendations (Fig. 1). On average, military experts submitted slightly less points (-1.8 points) and allowed more time (26 s more) across all medical interventions. Opinions varied more in the civilian versus the military group with respect to time goals (4.5 versus 4.0 min in average standard deviation, respectively). Conversely, military expert opinion varied more with respect to point scoring with an average standard deviation of 6.1 points versus 5.1 points for civilian experts.

Removal of weapons and dangerous objects was variably weighted among the experts. Civilian experts considered this aspect highly important and unanimously assigned it the maximum score. In contrast, military experts deemed this component less important with a lower average score of 19.9 points, albeit with substantial variability (SD 10.9).

Further, military experts advocated for less time allowance to treat the three exsanguinating hemorrhages with a mean of 1.8 ± 0.8 min, while civilian experts allowed 3.9 ± 3.1 min. Both expert groups ranked this treatment aspect as equally important with a mean of 27.6 points each (for the three bleeding sites). In contrast, an opposite time trend was observed for items in the circulation section, where civilian experts allotted less overall time than military experts (9.0 ± 4.6 versus 11.4 ± 4.6 min, respectively). Further, civilian experts weighted circulation interventions on average higher than their military colleagues (24.3 ± 4.3 versus 20.8 ± 6.4 points, respectively). Specifically, more importance was placed on pelvic binder application, transfusion management and assessing for other bleeding sites.

Neurologic assessment was rated lower in the military versus civilian expert group (18.4 ± 5.6 versus 22.2 ± 5.1 points), respectively. This difference was primarily driven by lower weighting of assessing for Glasgow Coma Scale (16.1 ± 6.9 versus 25.1 ± 5.0 points) and peripheral neurological deficits (13.3 ± 7.3 versus 20.0 ± 6.5 points) by military versus civilian experts, respectively (See Fig. 1; Table 2).

**Table 2:** Comparison of summarized ratings between military and civilian experts

	Time Army ± SD [min]	Importance Army ± SD [Points]	Time Civil ± SD [min]	Importance Civil ± SD [Points]	Total Time ± SD [min]	Total Importance ± SD [Points]
**General (group ø)**	**7.2 ± 4.0**	**21.6 ± 7.0**	**6.0 ± 3.9**	**25.3 ± 4.0**	**6.6 ± 4.1**	**21.7 ± 6.3**
Removal of arms	1.9 ± 1.5	19.9 ± 11.0	1.9 ± 1.6	30.0 ± 0.0	1.9 ± 1.5	24.9 ± 9.1
Full body examination	6.9 ± 4.0	24.6 ± 3.6	7.0 ± 3.8	24.6 ± 4.6	6.9 ± 3.8	24.6 ± 4.0
Primary Survey	8.3 ± 2.2	26.1 ± 3.5	3.7 ± 1.8	28.6 ± 3.8	6.0 ± 3.1	27.4 ± 3.7
Checking relevant vitals	9.9 ± 5.2	23.6 ± 6.9	5.4 ± 4.3	27.9 ± 2.7	7.6 ± 5.1	25.7 ± 5.5
Hypothermia control	7.6 ± 4.4	21.9 ± 8.8	7.9 ± 5.4	19.3 ± 6.1	7.7 ± 4.7	20.6 ± 7.4
Log-roll	8.9 ± 6.4	13.6 ± 8.0	10.0 ± 6.5	21.7 ± 7.1	9.4 ± 6.2	17.6 ± 8.4
Antisepsis		8.6 ± 2.4		14.3 ± 7.3		11.4 ± 6.0
**Exsanguinating Bleed (group ø)**	**1.8 ± 0.8**	**27.6 ± 4.5**	**3.9 ± 3.1**	**27.6 ± 4.5**	**2.9 ± 2.4**	**27.6 ± 4.3**
Massive bleed left thigh	1.4 ± 0.5	27.9 ± 3.9	3.1 ± 2.5	28.0 ± 3.8	2.3 ± 2.0	27.9 ± 3.7
Massive bleed right thigh	1.7 ± 0.8	27.9 ± 3.9	3.9 ± 3.1	27.9 ± 3.9	2.8 ± 2.4	27.9 ± 3.8
Massive bleed right upper arm	2.3 ± 1.1	27.1 ± 5.7	4.7 ± 3.7	27.0 ± 5.7	3.5 ± 2.9	27.1 ± 5.5
**Airway (group ø)**	**4.4 ± 3.6**	**22.0 ± 5.7**	**5.1 ± 4.2**	**23.5 ± 5.5**	**4.8 ± 4.2**	**22.8 ± 5.9**
Patient position	2.1 ± 0.9	23.0 ± 4.8	5.9 ± 5.0	19.0 ± 9.4	4.0 ± 3.9	21.0 ± 7.5
Surgical airway		27.9 ± 3.9		29.1 ± 2.3		28.5 ± 3.2
Verification of surgical airway	4.1 ± 2.4	25.0 ± 7.6	4.6 ± 2.7	27.0 ± 4.0	4.4 ± 2.5	26.0 ± 6.0
C-spine immobilisation	6.9 ± 7.6	12.1 ± 6.4	5.0 ± 5.0	18.9 ± 6.4	5.9 ± 6.3	15.5 ± 7.0
**Breathing (group ø)**	**9.6 ± 4.1**	**20.2 ± 6.3**	**9.6 ± 4.4**	**22.5 ± 5.6**	**9.6 ± 4.4**	**21.3 ± 6.3**
Clinical inspection of thorax	7.7 ± 5.1	20.0 ± 8.5	7.1 ± 4.7	23.7 ± 5.3	7.4 ± 4.7	21.9 ± 7.1
Supplemental oxygen	6.1 ± 2.0	22.9 ± 2.7	3.9 ± 5.1	27.1 ± 5.7	5.0 ± 3.9	25.0 ± 4.8
Medical imaging thorax	13.9 ± 6.6	14.3 ± 7.9	15.0 ± 2.9	16.4 ± 4.8	14.4 ± 4.9	15.4 ± 6.3
Basic therapy	7.1 ± 2.1	20.0 ± 4.1	7.1 ± 5.0	25.7 ± 5.3	7.1 ± 3.8	22.9 ± 5.4
Definitive surgical therapy	13.0 ± 4.6	24.0 ± 8.2	14.7 ± 4.5	19.3 ± 6.7	13.9 ± 4.5	21.6 ± 7.6
**Circulation (group ø)**	**11.4 ± 4.6**	**20.8 ± 6.4**	**9.0 ± 4.6**	**24.3 ± 4.3**	**10.2 ± 4.9**	**22.6 ± 5.9**
Re-evaluation massive bleeds	6.7 ± 4.9	25.0 ± 5.6	11.4 ± 3.8	22.7 ± 7.1	9.1 ± 4.9	23.9 ± 6.2
Diagnosing hemorrhagic shock	7.1 ± 4.6	26.0 ± 5.0	5.0 ± 4.7	29.0 ± 1.9	6.1 ± 4.6	27.5 ± 4.0
Pelvic binder	8.1 ± 3.5	18.6 ± 6.3	5.6 ± 4.4	26.9 ± 3.8	6.9 ± 4.1	22.7 ± 6.6
Peripheral venous access	9.4 ± 2.7	22.6 ± 8.8	5.6 ± 2.7	26.1 ± 3.5	7.5 ± 3.3	24.4 ± 6.7
Fluid resuscitation	12.4 ± 6.0	25.0 ± 5.0	8.3 ± 4.3	25.4 ± 5.1	10.4 ± 5.5	25.2 ± 4.9
Blood pressure management	16.4 ± 4.8	21.4 ± 6.3	9.6 ± 6.0	22.3 ± 4.1	13.0 ± 6.3	21.9 ± 5.1
Correct pRBC transfusion	15.0 ± 6.5	13.6 ± 7.5	13.4 ± 7.1	20.7 ± 5.3	14.2 ± 6.6	17.1 ± 7.3
Examination for other bleeds	16.3 ± 3.7	14.6 ± 6.9	12.9 ± 3.9	21.4 ± 3.8	14.6 ± 4.1	18.0 ± 6.4
**Disability (group ø)**	**11.7 ± 4.7**	**18.4 ± 5.6**	**9.5 ± 5.3**	**22.2 ± 5.1**	**10.6 ± 5.1**	**20.3 ± 5.8**
Register GCS	8.3 ± 5.2	16.1 ± 6.9	5.3 ± 3.6	25.1 ± 5.0	6.8 ± 4.5	20.6 ± 7.4
Peripheral neurological examination	14.1 ± 6.1	13.3 ± 7.3	10.4 ± 6.0	20.0 ± 6.5	12.3 ± 6.1	16.6 ± 7.5
Adequate analgesia	12.7 ± 2.9	22.6 ± 4.4	12.9 ± 6.4	21.3 ± 3.6	12.8 ± 4.7	21.9 ± 4.0
Anesthesia				21.4 ± 3.8		
**Management of coagulation (group ø)**	**11.2 ± 5.6**	**17.0 ± 6.2**	**10.9 ± 6.3**	**18.0 ± 5.9**	**11.0 ± 5.7**	**17.5 ± 5.9**
Tranexamic acid	9.7 ± 4.3	19.7 ± 6.0	9.0 ± 5.8	21.1 ± 5.8	9.4 ± 4.9	20.4 ± 5.7
Ca-Gluconate	12.0 ± 6.5	17.9 ± 5.7	11.6 ± 6.8	17.1 ± 3.9	11.8 ± 6.4	17.5 ± 4.7
Fibrinogen	11.9 ± 6.0	13.6 ± 6.9	12.1 ± 6.4	15.7 ± 7.9	12.0 ± 5.9	14.6 ± 7.2
**Other (group ø)**	**15.6 ± 4.3**	**16.2 ± 8.3**	**17.9 ± 3.9**	**15.4 ± 7.5**	**16.8 ± 4.2**	**15.8 ± 7.8**
Burn dressing	14.1 ± 4.7	15.0 ± 6.5	17.9 ± 3.9	17.1 ± 7.6	16.0 ± 4.6	16.1 ± 6.8
Antibiotic prophylaxis	17.1 ± 3.9	17.4 ± 10.2	17.9 ± 3.9	13.6 ± 7.5	17.5 ± 3.8	15.5 ± 8.8

## Discussion

This study aimed to develop a standardized assessment tool and evaluation framework for an upcoming in-situ, interprofessional, high-fidelity combat trauma simulation. Existing instruments were deemed insufficient to properly assess the critical principles of early management for penetrating, multisystem injuries relevant to both military and civilian practice, particularly with respect to clinical depth and contextual specificity. To address this, a multidisciplinary expert panel was convened, and a two-step consensus process was employed: an initial Nominal Group Technique (NGT) to generate and structure the preliminary framework, followed by a structured Delphi process to achieve consensus on quality metrics and target time frames for essential medical interventions. By combining these two consensus methods with a balanced selection of experts and a high degree of standardization -particularly regarding scoring rules and point deductions- we developed a robust, widely supported, and easily applicable tool that enables objective comparison across highly complex simulations. Additional standardizations, including post-hoc video analysis, the use of multiple raters, and structured rater debriefings, further minimize interrater variability. This framework is generalizable and can be applied to other simulations involving penetrating multisystem trauma.

Expert discussions revealed notable differences in early trauma care priorities between military and civilian healthcare providers, as well as between prehospital and in-hospital settings. These differences reflect the influence of guideline-driven frameworks such as ATLS^®^, PHTLS^®^, and TCCC^®^. A representative example from our consensus process highlights this divergence: military experts assigned shorter target times for initial management of massive hemorrhage compared to civilian experts (1.8 ± 0.8 vs. 3.9 ± 3.1 min). The “M” component of the military MARCH algorithm reflects operational experience/training with penetrating and blast-related injuries, where immediate control of life-threatening hemorrhage is critical for survival. In contrast, Swiss civilian trauma algorithms, largely shaped by routine exposure to blunt trauma [10], have historically placed greater emphasis on circulatory stabilization and bleeding control during the “C” phase of the ABCDE approach, with the “X” for exsanguinating hemorrhage only formally incorporated into the X-ABCDE algorithm in the 2023 ATLS^®^ update [21]. Prioritizing bleeding over airway management in exsanguinating injuries “CAB approach” follows the same principle and has been associated with improved survival compared with the traditional “ABC approach” [22]. This divergence highlights how professional background, operational context, and exposure to different injury patterns influence the perceived urgency and prioritization of specific life-saving interventions.

Initial fluid resuscitation was one of the most extensively debated topics during the Delphi process. In Central Europe, crystalloids are currently predominantly used during the prehospital and very early phase of polytrauma management, both in blunt [23] and penetrating trauma [24]. This is, among other factors, attributable to the recommendations of the 2023 European guideline on the management of major bleeding and coagulopathy following trauma, which still recommends the use of crystalloid solutions—preferably balanced solutions over normal saline—for the initial management of hemorrhagic shock (Grade 1B) [25]. In addition, economic considerations, logistical constraints, and short transport times with rapid access to trauma centers capable of providing transfusion support mean that most prehospital emergency medical services do not carry blood products and instead prioritize rapid transport (“load-and-go” strategy). Furthermore, current trends in trauma resuscitation demonstrate even a shift toward more restrictive strategy, permissive hypotension, and the judicious use of vasopressors [24, 26]. 

In contrast, Tactical Combat Casualty Care (TCCC) guidelines have largely moved away from crystalloid resuscitation altogether, instead advocating for early administration of whole blood (WB) or blood components (1:1:1 pRBC: FFP: platelets) in early treatment of hemorrhagic shock [6]. These recommendations are largely based on the 2024 guidelines of the American College of Surgeons Committee on Trauma, which recommend crystalloids only when blood products are unavailable [27]. The reasons why WB transfusion remains the treatment of choice for trauma-induced hemorrhagic shock in the military setting are not solely medical [28]. WB offers substantial logistical advantages, particularly regarding transport, storage, and rapid availability in austere environments [29]. In addition, fresh WB can be obtained from pre-screened personnel through so-called “walking blood banks,” significantly improving access during mass-casualty incidents or when stored blood products are limited [30]. Within the Swiss Armed Forces, a program for pre-screened low-titer group O fresh whole blood (F-LTOWB) exists; however, the use of cold-stored low-titer whole blood (CS-LTOWB) is currently not feasible due to the lack of supporting civilian infrastructure and regulatory constraints.

From a medical perspective, the evidence for the use of WB remains heterogeneous; [31] however, emerging data suggest potential early and late survival benefits compared with the predominant use of crystalloids [28, 32] as well as an increasing trend toward superiority over blood component therapy in civilian [33]—but not military—settings [28, 34]. Further research is clearly required; however, several ongoing studies (TROOP, WEBSTER) are expected to provide greater clarity on this central issue in trauma care in the coming years. In many Central European countries including Switzerland, component therapy remains the predominant transfusion strategy, while structured WB programs and experience with CS-LTOWB are limited. Notable exceptions include countries such as Norway or UK, where WB has been successfully implemented in both prehospital [31] and in-hospital settings for several years [35]. In Switzerland, where this study is planned, none of the participating prehospital organizations has access to whole blood or blood component therapy in the prehospital setting. In light of current evidence, local logistical and economic constraints, and the simulated early stabilization phase with a strictly limited 25-minute timeframe, the expert panel defined fluid resuscitation according to institutionally available and supported local recommendations/guidelines, ranging from crystalloid therapy to hospital-based massive transfusion protocols or packed red blood cells (pRBCs) alone. This consensus was made despite evidence suggesting superiority of whole blood or a balanced component strategy (optimally a 1:1:1 pRBC: FFP: platelets) transfusion strategy, over alternative resuscitation ratios [27]. 

An additional option for early volume resuscitation and coagulation optimization, particularly in military and austere prehospital settings, is lyophilized (freeze-dried) plasma. Its advantages are primarily logistical, including storage at room temperature, long shelf-life, and rapid reconstitution (2–6 min). From a clinical perspective, plasma-based resuscitation may provide survival benefit in patients with prolonged prehospital times (> 20 min) compared with standard resuscitation strategies, whereas benefits appear less pronounced in systems with rapid access to blood products and definitive trauma care [36, 37]. With respect to coagulation management, regional practice variation exists, with North American systems predominantly using ratio-based plasma resuscitation, while European centers increasingly rely on coagulation factor concentrates guided by viscoelastic testing (ROTEM/TEG) [38]. However, in the context of the present assessment framework, lyophilized plasma was discussed but not incorporated into scoring, primarily due to its limited availability across participating institutions and prehospital systems.

In contrast to fluid resuscitation, there was broad consensus regarding the administration of tranexamic acid (TXA). The benefit of early TXA in trauma-induced coagulopathy is well established [39]. Differences remain regarding dosing recommendations, with military protocols commonly advocating a total dose of two grams administered as an infusion or slow push, whereas civilian guidelines typically recommend an initial one gram bolus followed by a one gram infusion over eight hours. Current evidence does not conclusively favor one dosing strategy over the other, and the expert panel therefore considered both approaches acceptable within the evaluation framework [40]. 

Fibrinogen is known to be the first coagulation factor to fall to critical levels during massive hemorrhage, and early replacement has been associated with improved coagulation profiles [41]. However, uncertainty remains regarding the optimal timing of fibrinogen substitution, with current evidence favoring targeted correction based on point-of-care coagulation testing [42]. In the planned study setting, resource availability and institutional practices varied considerably. In addition, the preparation and administration of cryoprecipitate or fibrinogen concentrate is time-consuming and often not feasible within the 25-minute simulation window. Consequently, the expert panel elected to award bonus points for the use of point-of-care coagulation testing or the initiation of fibrinogen administration. This approach acknowledged the clinical relevance of coagulation optimization while ensuring that teams could still achieve a maximum score without mandatory factor substitution.

The removal of weapons and other hazardous objects represented another area of marked divergence between civilian and military perspectives. Civilian participants consistently rated this task as highly important, assigning the maximum score with minimal variability (30.0 ± 0), reflecting strong concerns regarding scene safety and institutional risk management. In contrast, military participants assigned a lower average importance (19.9 ± 10.9), likely reflecting greater familiarity with weapons handling and a higher tolerance for residual risk in combat environments. Notably, variability within the military group was substantial, suggesting that individual experience and operational role strongly influence prioritization. In active combat environments, military medics may leave a lightly injured combatant armed if not completely incapacitated, as they may still retain some operational capability. Strict indications for disarmament include clinically relevant traumatic brain injury with impaired consciousness, any form of shock, or administration of psychoactive substances.

In summary, the differences observed between civilian and military experts underscore the value of structured interdisciplinary dialogue when defining performance standards for combat trauma simulation. Rather than enforcing strict adherence to a single guideline framework, the evaluation tool was intentionally designed to reflect an optimal, guideline-informed performance envelope that accommodates differing operational contexts and realities. This approach enables the simulation to serve not only as an assessment instrument, but also as a platform for bidirectional learning and civilian–military knowledge exchange.

This study has several limitations. The initial Nominal Group Technique (NGT) was conducted with a smaller subset of experts for logistical reasons, which may have limited the opportunity for all experts to fully contribute despite opportunities to suggest adjustments during the Delphi rounds. The Delphi panel was intentionally limited in size, and although balanced between civilian and military experts, subgroup analyses should be interpreted as exploratory. Many recommendations are based on expert consensus rather than definitive empirical evidence, particularly in areas lacking high-quality randomized data. Additionally, the Delphi process spanned approximately 120 days, and some information may have been overlooked or forgotten over time. Finally, although the assessment tool reflects broad expert consensus, it has not yet undergone formal validation, and its robustness, reliability, and external generalizability remain to be established. Differences between military and civilian experts were described descriptively only and not subjected to formal significance testing due to the small sample size, as such analyses would not have added meaningful value to the consensus process.

## Conclusion

This Delphi-based evaluation framework highlights both areas of consensus and ongoing controversy in modern combat trauma care. Differences between civilian and military perspectives are largely driven by divergent injury patterns, resources, and operational constraints. By explicitly incorporating these differences into a standardized evaluation tool, this study provides a robust framework for our upcoming simulation study, and more importantly, for comparing performance across different levels of trauma care.

## Supplementary Information

Below is the link to the electronic supplementary material.

Supplementary Material 1: Appendix 1: Detailed description of the simulation scenario, including preparation and implementation guidelines, as well as safety considerations.

Supplementary Material 2: Appendix 2: Comprehensive overview of the Delphi rounds in both narrative and tabular format.

Supplementary Material 3: Appendix 3: Detailed ratings of importance/quality (scores from 1 to 30) and target times for completion of interventions (minutes), stratified by civilian and military experts.

## Data Availability

All data supporting the findings of this study are available within the paper and its Supplementary Information.

## Declarations

### Adherence to principles

The study was conducted in coordination and in accordance with the principles of the University Hospital of Zürich Simulation Center.

## Abbreviations

18D: Special Forces Medical Sergeant (US Army Education)
ATLS: Advanced Trauma Life Support
CAB: Circulation Airway Breathing Approach
CRM: Crew Resource Management
C-Spine: Cervical Spine
Ca-Gluconate: Calcium-Gluconate
CS-LTOWB: Cold-stored Low-titer Group O Whole Blood
FFP: Fresh Frozen Plasma
F-LTOWB: Pre-screened Low-titer Group O Fresh Whole Blood
GCS: Glascow Coma Scale
IED: Improvised Explosive Device
MEDEVAC: Medical Evacuation
NATO: North Atlantic Treaty Organisation
PHTLS: Prehospital Trauma Life Support
Prbc: Packed Red Blood Cells
ROTEM: Rotation Thromboelastometry
SD: Standard Deviation
TCCC: Tactical Casualty and Combat Care
TEG: Thromboelastography
NGT: Nominal Groupe Technique
TXA: Tranexamic Acid
WB: Whole Blood
