# Supplementary material for: A novel evaluation tool for combat trauma simulation: Delphi expert panel consensus report
Source: Patient Saf Surg. 2026 Jun 26;20:32. doi: 10.1186/s13037-026-00493-z (PMC13602610; doi:10.1186/s13037-026-00493-z)
Supplement: Supplementary file 1 [file 13037_2026_493_MOESM1_ESM.docx]

**Appendix 1**

**Development of a Case-Specific Performance Evaluation Tool for a Combat Trauma Simulation stud: A Delphi Consensus process report**

**Safety**

Training on a actor-patient using real medications, including opioids and hypnotics, as well as performing actual medical interventions on the actor such as emergency front-of-neck access, surgical procedures, vessel clamping, needle decompression, or finger thoracostomy, entails a significantly increased risk of real-world complications. To mitigate these risks, a comprehensive safety framework has been implemented. The safety measures include:

- A mandatory **safety briefing** prior to each simulation, during which all participants are clearly informed about permitted and prohibited medical interventions.
- A clearly defined **safe word**. In the military setting, the agreed command is **“No Play, No Play,”** which immediately halts the simulation.
- The continuous presence of a **safety supervisor** in the room whose sole responsibility is the safety and well-being of the actor. This supervisor is authorized to intervene at any time and is responsible for promptly releasing tourniquets, pressure dressings, or other potentially harmful measures.
- Continuous **radio communication** between the actor and the simulation director to ensure immediate reporting of discomfort, unexpected pain, or any safety concerns.

**Patient Handover**

Patient transport is conducted in accordance with common practice in the Swiss Armed Forces and many other military systems, using a barre (military stretcher) rather than a conventional civilian emergency stretcher. In the military setting, standard stretchers are often impractical and therefore not widely used. As a result, the transfer from a barre to a hospital emergency stretcher represents often the first unfamiliar situation for many clinical teams.

Handover is performed according to the military TCCC standard using the **MIST format** (Mechanism of injury, Injuries, Signs/Symptoms, Treatments). The handover is intentionally concise and efficient but includes the key injury patterns. Military personnel assist with patient transfer and then withdraw during the initial phase of the medical simulation.

For prehospital and military teams, patient handover is adapted to the tactical context and may occur either at transition points within the phases of care (e.g., Tactical Field Care/Tactical Evacuation Care) or upon transfer to a higher level of medical support. In all cases, handover follows standardized principles comparable to those used in the in-hospital setting.

**Simulated Injury Pattern**

The medical injury pattern reproduced in this simulation was based on original video footage from a U.S. military incident. The mechanism of injury was the detonation of an improvised explosive device (IED) combined with a booby-trap mechanism. The explosion was triggered upon stepping near the device, resulting in a blast with multiple metallic shrapnel fragments. According to the reference case, a significant proportion of the blast energy was directed toward the victim’s face and the left part of the body. Based on this template, the following complex injury pattern was recreated:

- **Severe** **maxillofacial trauma** associated with significant soft tissue loss, deep facial burns, and progressive airway obstruction.
- **Shrapnel entry** **wounds** at the left hemithorax, pelvis, and left thigh, without major external hemorrhage.
- **Active hemorrhage from shrapnel exit wounds**, including:
  - The left upper arm, with an open humeral fracture and active bleeding from both soft tissue and exposed bone.
  - The posterior aspect of the left thigh.
  - The gluteal region.
- **Left-sided** **hemato-pneumothorax**, resulting in progressive oxygenation impairment and contributing to an obstructive component of shock.
- **Extensive** **second- and third-degree burns** involving the left hemithorax, affecting approximately 20% of the total body surface area.
- **Suspected** **pelvic fracture**, secondary to the blast wave and subsequent fall.
- **Functional sensory impairment**, including acute loss of vision and hearing in the context of the facial trauma and blast exposure.
- **Mild to moderate traumatic brain injury (TBI)** due to the blast wave and subsequent fall.
- **Acute stress reaction**, reflecting the psychological and physiological impact of the explosion.

This constellation represents a highly complex combined injury scenario typical of modern combat trauma, characterized by the simultaneous presence of airway compromise, hemorrhagic shock, obstructive shock, burn injury, orthopedic trauma, and psychological stress.

**Medical Script**

**Craniofacial Trauma and Airway Problem**

Due to the direct exposure of the face to the explosive device (based on the reference case, in which the victim was running toward the device at the moment of detonation), a substantial proportion of the blast energy is transmitted directly to the craniofacial region. This results in extensive destruction of the mandible, maxilla, nasal structures, and surrounding soft tissues, including the eyes and ears. The injury is associated with severe soft tissue disruption and predominantly third-degree burns affecting the facial skin and adjacent tissues.

**Picture 5**, Massive craniofacial trauma during treatment of a military medical unit

***Picture 2,*** *Preparation of the craniofacial trauma of a simulation instructor*

**Initial Presentation and Dynamic Progression**

Upon first contact, the patient is conscious and able to maintain spontaneous airway patency independently. However, verbal communication is severely impaired; the patient can only produce incomprehensible sounds due to structural destruction of the oropharyngeal anatomy. Spontaneous breathing is present, with initially acceptable oxygenation depending on supplemental oxygen delivery.

The progression of airway obstruction depends strongly on patient positioning and early airway management. The underlying mechanisms are multifactorial: decreasing consciousness due to hemorrhagic shock leads to loss of protective airway reflexes and reduced ability to maintain airway patency, while inhalation of hot explosive gases causes thermal injury and progressive upper airway edema. Together, these factors result in a dynamically worsening airway compromise requiring early anticipation and intervention.

- If the patient is positioned supine and remains flat for the first 10 minutes, progressive pooling of blood and soft tissue collapse leads to complete airway obstruction after approximately 10 minutes. This manifests as an acute panic reaction, obstructive breathing sounds (gurgling/stridor), agitation, followed by rapid deterioration in oxygen saturation and level of consciousness.
- If the patient is managed with head-up elevation, intermittent lateral positioning, or regular suctioning of supraglottic secretions and blood, airway obstruction develops more slowly but remains progressive. In this case, critical obstruction occurs after approximately 20 minutes.

**Oxygenation and Non-invasive Measures**

During the phase of spontaneous breathing, supplemental oxygen via face mask or reservoir mask leads to immediate improvement in oxygen saturation. However, placement of nasopharyngeal, oropharyngeal, or supraglottic airway devices fails due to mechanical obstruction at the level of the destroyed maxilla and distorted upper airway anatomy. These interventions do not result in sustained respiratory improvement.

**Advanced Airway Management**

Endotracheal intubation, whether performed with conventional direct laryngoscopy or video-assisted laryngoscopy, is unsuccessful due to mechanical obstruction by fractured bone fragments and severe anatomical distortion preventing visualization and access to the larynx. Fiberoptic intubation also fails because of massive bleeding, disrupted anatomy, and visual field contamination.

A surgical airway (emergency front-of-neck access, eFONA) is feasible, as the anterior neck structures remain largely intact. When performed correctly and in a timely manner, the procedure is successful and results in immediate improvement in ventilation and oxygenation. Adequate tube placement with effective ventilation leads to rapid stabilization of respiratory parameters. If no local anesthesia or systemic analgesia is administered prior to skin incision, the patient demonstrates a withdrawal movement in response to the surgical stimulus, reflecting preserved nociceptive response and adding procedural complexity under stress conditions.

**Picture 7**, eFONA, Bougie-aided technique

**Picture 6**, eFONA (emergency front of neck access) on a real actor during a Test simulation

**Penetrating Injuries, Hemorrhage Control, and Hemodynamic Progression**

**Active External Hemorrhages**

The first major bleeding site is located at the left upper arm, where a large shrapnel fragment has caused extensive soft tissue destruction and an open humeral fracture. The predominant bleeding originates from both the exposed bone and the surrounding soft tissue injury. Hemorrhage control can be effectively achieved either by application of a sufficiently proximal tourniquet or by careful limb realignment followed by firm compression with a pressure dressing. Inadequate tourniquet placement (too distal or insufficiently tightened) results in persistent bleeding.

The second major bleeding source is found in the gluteal region, where a large shrapnel exit wound with a deep wound cavity is present. Hemostasis can be achieved by aggressive wound packing combined with sustained manual compression and a pressure dressing. Application of a pelvic binder may contribute to bleeding control if a concomitant pelvic fracture is suspected. Direct vascular clamping within the depth of the wound cavity is technically possible but requires deliberate exploration and carries procedural complexity.

The third active bleeding site is located at the posterior aspect of the left thigh, corresponding to another shrapnel exit wound. Management options include wound packing with pressure dressing or tourniquet placement.

**Picture 8**, Open humeral fracture with hemorrhage controlled by tourniquet application, previously loosened by an instructor on the live actor for safety reasons.

**Picture 3,** Attempted Wound Packing of the Large Gluteal Exit Wound

**Hemodynamic progression**

If not all active bleeding sources are identified and adequately controlled, the patient progressively develops hemorrhagic shock. Clinically, this is reflected by increasing tachycardia, hypotension, delayed capillary refill, pallor, and deteriorating mental status. From a hemodynamic perspective, ongoing blood loss results in persistent instability despite supportive measures. Isolated volume resuscitation or vasopressor therapy in the presence of uncontrolled hemorrhage has only minimal and transient effects on clinical status and vital parameters. Blood pressure may show brief improvement but rapidly deteriorates again as bleeding continues. This aspect of the scenario is designed to reinforce the principle of “control the bleeding first” in accordance with damage control resuscitation strategies.

Sustainable hemodynamic stabilization can only be achieved through a combination of effective hemorrhage control, appropriate volume replacement, and, if necessary, adjunctive vasopressor therapy. Transfusion of two units of packed red blood cells results in rapid and clinically significant stabilization of circulation. Alternatively, short-term bridging with crystalloids or colloids (>1 liter) may temporarily improve hemodynamics. This component of the scenario emphasizes prioritization within the “XABCDE approach”, the importance of systematic bleeding search, and the limited efficacy of pharmacologic or volume-based interventions in the absence of definitive hemorrhage control.

**Thoracic Injury, Internal Bleeding, and Obstructive Shock Component**

**Internal Bleeding**

Due to an additional shrapnel injury to the left thorax with both entry and exit wounds, the patient progressively develops a left-sided hemato-pneumothorax. The clinical course is highly dependent on the interventions performed.

As long as the airway remains patent and the patient continues spontaneous breathing, respiratory function deteriorates gradually over the first 15 minutes. This is characterized by increasing dyspnea, tachypnea, reduced breath sounds on the left side, and progressive impairment of oxygenation. If the condition is recognized early based on clinical findings (unilateral decreased breath sounds, respiratory distress, hypoxia), point-of-care ultrasound, or radiographic imaging and treated appropriately, rapid stabilization can be achieved. Effective interventions include application of a chest seal (spontaneous breathing of the patient) for an open chest wound and decompression of the tension component via needle decompression or finger thoracostomy. Timely decompression leads to prompt improvement in respiratory mechanics and oxygenation.

However, if positive-pressure ventilation is initiated without prior or immediate decompression of the tension component, intrathoracic pressure rises rapidly. Within 3–5 minutes, the patient develops signs of obstructive shock, including worsening hypotension, tachycardia, distended neck veins and rapidly declining oxygenation. Without intervention, this progression leads to near-cardiac arrest physiology. This element of the scenario is designed to reinforce early recognition of tension physiology, the importance of prioritizing decompression before or immediately after initiation of positive-pressure ventilation, and the dynamic interaction between respiratory management and hemodynamic stability in penetrating thoracic trauma.

**Picture 10**, Open surgical chest tube insertion performed on a simulator placed on the actor-patient.

**Picture 9**, Open sucking chest wound of the left hemithorax.

**Associated Injuries**

**Burn Trauma**

In addition to the penetrating and blunt injuries described above, the patient presents with extensive burn injuries. Second- and third-degree burns are present, with fourth-degree burns affecting parts of the face. The total body surface area (TBSA) involved is approximately 20%. The burns are distributed across the face, the left hemithorax, and the left lower extremity, particularly the lower leg. The facial burns are associated with deep tissue destruction and contribute significantly to airway compromise and soft tissue instability. During the initial phase represented in the simulation—namely the first 30–60 minutes of care—the hemodynamic impact of burn shock is of minor clinical relevance. In this early time window, the dominant driver of circulatory instability is hemorrhagic or obstructive shock. Adequate management of bleeding and volume resuscitation effectively compensates for the early burn-related fluid shifts. This component of the scenario is intended to highlight the need for early burn assessment and appropriate planning (e.g., estimation of TBSA, prevention of hypothermia), while emphasizing that, in the acute phase of combined blast trauma, hemorrhage control remains the primary life-saving priority.

**Pelvic Fracture and Psychological Impact**

In addition, the patient sustains a pelvic fracture, a common injury pattern in blast mechanisms and subsequent fall events. The immediate clinical consequence is an additional internal source of hemorrhage, potentially leading to catastrophic blood loss of up to 3 or more liters. This significantly aggravates the already existing hemorrhagic shock. If no pelvic binder or alternative mechanical stabilization is applied within the first 10 minutes, ongoing pelvic bleeding contributes to progressive hemodynamic deterioration and increasing refractoriness to resuscitative measures. In this context, volume therapy and vasopressors alone remain insufficient unless mechanical stabilization and hemorrhage control are achieved. Early recognition and prompt application of a pelvic binder are therefore critical components of damage control management in this scenario.

Beyond the physiological consequences, the patient also exhibits signs of acute psychological distress. Blast-related loss of vision and hearing—particularly in military personnel—represents a dramatic and disorienting experience. The sudden sensory deprivation may manifest as agitation, panic, and emotional instability. This acute stress reaction can further complicate clinical management, as it may distract the treatment team, increase cognitive load, and challenge structured decision-making. This aspect of the scenario is intended to highlight not only the need for systematic examination an treatment, but also the importance of maintaining team focus and situational awareness in the presence of emotionally distressing patient factors.

**Patient Death Within the Simulation**

Balancing maximal realism with educational benefit and psychological safety, we deliberately decided that the patient would not die during the simulation under any circumstances. We are fully aware that, from a medical perspective, uncontrolled hemorrhage within the first 30 minutes of care would almost inevitably lead to death or to an irreversible, non-survivable state of hemorrhagic shock. However, early termination of the scenario due to simulated patient death was considered to carry substantial educational disadvantages. Premature scenario termination would limit experiential learning, reduce opportunities for corrective action, and potentially compromise psychological safety within the team.

Instead, failure to identify and adequately control bleeding, insufficient management of obstructive or hemorrhagic shock, or persistent untreated airway obstruction results in a clearly defined peri-arrest state within the simulation. This is represented clinically and through monitored vital signs by absent peripheral pulses, profound hypotension (systolic blood pressure approximately 30–40 mmHg), and signs of imminent circulatory collapse. This approach preserves medical plausibility while allowing teams to recognize critical deterioration, reflect on decision-making processes, and implement corrective measures without abrupt scenario termination.

**Preparation of the Simulators**

**eFONA on a Actor-Patient**

For the performance of emergency front-of-neck access (eFONA) on a live actor, a purpose-built simulation model was developed to maximize procedural realism while ensuring full actor safety. A 3D-printed laryngeal framework was firmly mounted onto a prefabricated carbon protective plate (**Picture 11**), which serves as a rigid barrier protecting the actor’s neck. The cricothyroid membrane was simulated using a reinforced adhesive layer designed to reproduce realistic tactile resistance during incision. The entire assembly was then covered with a multilayer artificial skin system replicating epidermis, subcutaneous tissue, and deeper soft tissue structures, thereby providing authentic haptic feedback during cutting and dissection.

To further enhance fidelity, approximately 3–5 ml of artificial blood can be injected into the tissue layers prior to the procedure (**Picture 12**), producing visible and realistic bleeding upon incision. In addition, a small artificial blood clot is placed within the simulated tracheal lumen to mimic intraluminal blood contamination, thereby increasing procedural complexity and reflecting real-world airway conditions in severe maxillofacial trauma. This setup enables highly realistic simulation of surgical airway management while maintaining complete physical protection of the actor at all times.

**Picture 13**, Fully assembled wearable model that can be securely fitted and worn by the actor-patient.

**Picture 12**, Artificial multilayer skin with integrated capability for injection of simulated blood.

**Picture 11**, Carbon protective plate with 3D-printed model of a larynx

**Bleeding Simulators**

The bleeding functionality of the moulages/sleeves was integrated directly onto the actor-patient. The individual bleeding modules were worn by the actor, and the attached plastic tubing was routed along the body and secured with skin-colored adhesive tape. All tubes were then converged and externally routed at the posterior neck. Each individual tube was connected to a centrally controlled manual pump system. This setup allowed selective simulation control: by disconnecting a specific tube, hemorrhage from the corresponding wound could be stopped to simulate successful bleeding control; reconnection reinitiated active bleeding. All tubing was explicitly marked as simulation equipment, and the participating teams were informed about its presence during the pre-briefing to maintain transparency and psychological safety. The manual pump was connected to a concealed reservoir containing approximately 3 liters of artificial blood. The reservoir and pump system were camouflaged within a small tactical pouch worn by the patient, ensuring functional realism while maintaining operational control of the simulated hemorrhage.

**Picture 15** All plastic tubing and radio connections are routed centrally at the back of the actor-patient’s neck and clearly marked as simulation equipment with blue tape.

**Picture 14**, Additional room to prepare the actor-patient and to set up the moulages/sleeves.

**Thoracic Interventions and Vascular Accesses**

For the performance of surgical thoracic interventions, we opted to use an external torso model to represent the full range of thoracic procedures. A portable thoracic simulator was also tested; however, due to its limited capabilities and frequent dislocations during use, it was not adopted.

For intravenous (IV) and intra-arterial (IA) access, an IV simulator with an integrated carbon protective plate was attached to the actor’s right upper extremity. This setup provides realistic haptic and visual feedback for vein puncture, and any fluids or medications administered are collected via tubing into a central reservoir. Additionally, the simulation leader can attach a separate IV or IA pad to any desired anatomical location on the actor if additional access points are required. Placement of central venous catheters or vascular sheets was prohibited in the simulation for both safety and time constraints, and because they were considered of limited clinical relevance in this scenario; this was clearly communicated during the safety briefing.

**Picture 17**, IV simulator attached directly to the actor-patient, alongside an additional modular simulator that can be flexibly applied to the affected body site during the simulation.

**Picture 16**, A multifunctional torso in use, allowing the performance of a range of surgical procedures.

**Simulators Used**

Maxilofacial Trauma	Battlefield Partners Inc., CRIC MASK, Massive maxillofacial trauma

Humeral fracture	Battlefield Partners Inc., Bicep Sleeve - Severe Fracture

Exit wound gluteal	Battlefield Partners Inc., Advanced Lower Body - Gunshot Wound

Exit wound leg	Battlefield Partners Inc., Tight Sleeve – Gunshot wound

IV Simulator 1	Battlefield Partners Inc., Arm Sleeve – IV Sleeve with Carbon Armour

Veins	Battlefield Partners Inc., Replacemeint Veins

IV/IA Simulator 2	Erler Zimmer, IV Trainer

Bleeder	Battlefield Partners Inc., Easy Bleeder Manual Backpack

Torso	Erler Zimmer, Thorax drainage Simulator

Burns/Torso Injuries	Erler Zimmer, Wounds – for Simulation patient

Multilayer skin	Battlefield Partners Inc., Bleeding Strips Replacement

Make-up	Erler Zimmer, Wound Simulationset “Professional”
