# Supplementary material for: A novel evaluation tool for combat trauma simulation: Delphi expert panel consensus report
Source: Patient Saf Surg. 2026 Jun 26;20:32. doi: 10.1186/s13037-026-00493-z (PMC13602610; doi:10.1186/s13037-026-00493-z)
Supplement: Supplementary file 2 [file 13037_2026_493_MOESM2_ESM.docx]

**Appendix 2,** **Comprehensive Overview of the Delphi Rounds**

**Development of a Case-Specific Performance Evaluation Tool for a Combat Trauma Simulation study: A Delphi Consensus process report**

**Summary**

Overall Rounds: 5

Overall Duration: 120 days

During the Delphi process, multiple content adaptations were made to the preliminary assessment framework developed from the NGT, focusing on fluid resuscitation, coagulation management, thoracic injury, analgesia/anesthesia, transfusion, and surgical airway interventions. Disagreements were carefully discussed, leading to standardized definitions, scoring rules, and bonus points where appropriate.

**Fluid Resuscitation:**

- Military experts emphasized avoiding crystalloids (especially 0.9% NaCl) due to dilutional coagulopathy. Civilian/pre-hospital experts stressed availability and initial stabilization efficacy.
- Resolution: Fluid therapy assessed according to institutional SOPs, including both blood products and crystalloids (NaCl or balanced solutions). Point deductions for incorrect administration were removed.

**Early Coagulation Management and Tranexamic Acid:**

- TXA dosing standardized: Military 2 g IV bolus, Civilian 1 g IV bolus or short infusion.
- Bonus points (15 points) awarded if a team correctly performs point-of-care coagulation testing and/or administers fibrinogen (empirical 2–6 g IV) within the simulation.
- Calcium gluconate (2.25 mmol) included in pRBC handling scoring.

**Thoracic Injury Management:**

- Basic therapy: occlusive dressing, chest seal, needle decompression. Correct application: max points; −5 pts for errors (improper chest seal, wrong decompression site, incorrect needle use).
- Advanced surgical therapy: Chest drain, finger thoracostomy, thoracotomy; –5 pts per error: disinfection, local anaesthesia/anaesthesia, correct localization, correct surgical technique, correct fixation, position check

**Analgesia and Anesthesia:**

- Analgesia: Fentanyl 100–150 μg IV / 400–800 μg PO, Ketamine 25–50 mg IV. −5 pts for underdosing, −10 pts for overdosing.
- Anesthesia: Midazolam 5–15 mg IV, Propofol 150–300 mg/hour IV, Sevoflurane < MAC 0.7. −5 pts for underdosing, −10 pts for overdosing.

**Transfusion Management:**

- Correct handling of pRBCs: ABO check, visual inspection, expiry date, transfusion set use, crossmatch if indicated, reassessment. Administration of 2.25 mmol calcium gluconate included. Errors result in −3 points per mistake.

**Surgical Airway:**

- Surgical cricothyrotomy: no fixed time goal due to patient positioning and scenario variability. Only time for subsequent patient position check scored.

**Detailed Round-by-Round Delphi Deliberations**

| **Round** | **Focus / Actions** | **Key Discussion Points** | **Decisions / Adaptations** |
| --- | --- | --- | --- |
| **Round 1** | Initial quantitative rating of all NGT-derived items (importance/importance 1–30 points; target time for interventions) | - Base therapy for thoracic injuries insufficiently described  - No time targets for surgical airway  - Unclear abbreviations  - Transfusion management vague  - Analgesia & anesthesia not precisely defined  - TXA dosing unclear  - Fluid therapy poorly defined | - Identified all issues for revision in Round 2 |
| **Round 2** | Review of Round 1 results of the quantitative rating (anonymized) | - Hemorrhage control recognized as most critical, multiple civilian experts rated fluid therapy as more important than hemorrhage control  - If TXA should administered per institutional SOP  - If Analgesia & anesthesia dosages should be standardized  - Surgical cricothyrotomy: no time goal as airway compromise is scenario dependant  - Fluid therapy debated: NaCl 0.9% vs. balanced crystalloids vs. blood  - Thorax base therapy should be defined exactly: occlusive dressing, needle decompression is considered as basic therapy  - Transfusion management refined, minimum for pRBC handling: ABO check, use of filter, documentation  - POC coagulation testing debated; retained for bonus points | - 2 Civilian raters increased importance of Hemorrhage control (15 to 30 points and 20 to 30 points)  - Exact definition of the item Analgesia: Fentanyl 100–150 µg IV / 400–800 µg PO; Ketamine 25–50 mg IV (-5 pts underdose, -10 pts overdose)  - Exact definition of the item Anesthesia: Midazolam 5–15 mg IV, Propofol 150–300 mg/h IV, Sevoflurane < MAC 0.7 (-5/-10 pts)  - TXA dosing adjustments is dependant of the institution (Civilian 1g, Military 2g)  - Thorax base therapy separated from advanced procedures  - Exact definition of the item Basic therapy thoracic injury: Occlusive dressing, chest seal, needle decompression; Max points: Correct application of a chest seal or similar device and, if indicated, needle decompression; −5 pts for clear errors (e.g., improper chest seal fixation, incorrect decompression site, or improper needle use).  - Exact definition of the item Advanced surgical therapy of thoracic injury: Chest drain, finger thoracostomy, thoracotomy; –5 pts per error: disinfection, local anaesthesia/anaesthesia, correct localization, correct surgical technique, correct fixation, position check |
| **Round 3** | Further refinements and agreement | - Fluid resuscitation: allow balanced crystalloids & blood; penalize NaCl  - TXA doses accepted (Civilian 1g, Military 2g)  - Analgesia & Anesthesia accepted  - Thorax base therapy accepted  - No time targets for surgical airway  - Calcium evidence limited; mainly combined with RBC transfusion  - Fibrinogen & POC coagulation: some clinicians insist on inclusion | - Fibrinogen / POC retained for bonus points pending literature review  - Fluid resuscitation: allow balanced crystalloids & blood; penalize NaCl (-10 pts) |
| **Round 4** | Further refinements and agreement | - Fluid therapy: NaCl 0.9% and balanced crystalloids acceptable per European Bleeding Management Guidelines 2023; point deduction removed  - Calcium integrated into pRBC handling (-3 pts per error) - Literature review for fibrinogen: early substitution <30 min not feasible in 25-min scenario  - Bonus points (15 pts) for POC or empirical 2–6 g fibrinogen correct administration | - Fluid therapy: follow institutional SOP including NaCl or balanced solutions  - Calcium included in RBC handling  - Fibrinogen eligible for bonus points; no mandatory time score |
| **Final Round** | Consolidation and final approval | - Circulated summary of all adaptations  - All experts reviewed remaining points | - Consensus reached: Calcium included in pRBC handling (-3 pts per error)  - Fibrinogen bonus points accepted (2–6 g IV or POC-guided)  - Final evaluation form formally approved by all 14 experts beyond the current study. |
